# Lung Cancer Risk from Occupational and Environmental Radon and Role of Smoking in Two Czech Nested Case-Control Studies

**DOI:** 10.3390/ijerph10030963

**Published:** 2013-03-07

**Authors:** Ladislav Tomasek

**Affiliations:** National Radiation Prtotection Institute, Bartoskova 28, Prague, Czech Republic; E-Mail: ladislav.tomasek@suro.cz; Tel.: +420-226-518-267; Fax: +420-241-410-215

**Keywords:** lung cancer, radon, smoking

## Abstract

The aim of the present study was to evaluate the risk of lung cancer from combined exposure to radon and smoking. Methodologically, it is based on case-control studies nested within two Czech cohort studies of nearly 11,000 miners followed-up for mortality in 1952–2010 and nearly 12,000 inhabitants exposed to high levels of radon in homes, with mortality follow-up in 1960–2010. In addition to recorded radon exposure, these studies use information on smoking collected from the subjects or their relatives. A total of 1,029 and 370 cases with smoking information have been observed in the occupational and environmental (residential) studies, respectively. Three or four control subjects have been individually matched to cases according to sex, year of birth, and age. The combined effect from radon and smoking is analyzed in terms of geometric mixture models of which the additive and multiplicative models are special cases. The resulting models are relatively close to the additive interaction (mixing parameter 0.2 and 0.3 in the occupational and residential studies, respectively). The impact of the resulting model in the residential radon study is illustrated by estimates of lifetime risk in hypothetical populations of smokers and non-smokers. In comparison to the multiplicative risk model, the lifetime risk from the best geometric mixture model is considerably higher, particularly in the non-smoking population.

## 1. Introduction

For a century, it has been known that some underground miners suffered from higher rates of lung cancer than the general population. In recent decades, a growing body of evidence has causally linked their lung cancers to exposure to high levels of radon and also to cigarette-smoking. The connection between radon and lung cancer in miners has raised concern that radon in homes might be causing lung cancer in the general population, although the radon levels in most homes are much lower than those found in most mines [1]. In the Czech Republic, two large cohort studies of lung cancer in relation to radon exposure, in uranium miners and in the general population were initiated by the late Josef Sevc. The results of the uranium miners study have been reported since 1971 [2]. Results of the cohort study extended to nearly 10,000 uranium miners were reported most recently in 2012 [3]. On the other hand, the environmental (residential) study in a region with relatively high levels of radon was established much later and first results were reported in 2001 [4], and most recently in 2012 [5]. As the risk of lung cancer depends both on the exposure to radon and its progeny and also on smoking, the simultaneous evaluation of the two carcinogens is substantial. Historically in studies of uranium miners, the effect of smoking was not evaluated in the first study, as the individual smoking information was not collected when the cohort was set up. However, such information was gathered later using the method of case-control study nested within the cohort. The aim of the present report is to evaluate the contribution of radon and smoking exposure to the risk of lung cancer in the occupational and residential radon studies. Epidemiologically, the combined risk from radon and smoking can be considered as additive or multiplicative. In this report, the risk is evaluated using the approach of geometric mixture models suggested in BEIR VI report [1], where the additive or multiplicative models are special cases.

## 2. Methods

### 2.1. Study Populations

The present studies are case-control studies nested within several cohort studies described below with follow-up ending at the end of 2010. The study population of the occupationally exposed cohort consists of a total of 10,890 miners exposed in uranium and claystone mines. The older sub-cohort (*S*) involves 4,352 uranium miners from the Jachymov region in west Bohemia, who began underground work in the period 1948–1959 and worked for at least four years [3]. The newer sub-cohort (*N*) consists of 5,626 uranium miners who entered the Pribram mines in central Bohemia during the period 1968–1974, when hygienic measures had been already fully implemented [3,6]. The miners selected for this sub-cohort worked for at least one year. The third sub-cohort (*L*) consists of 912 claystone miners exposed at least for a month in the period 1960–1980 [6].

The residential study was designed in 1989 as a retro-prospective follow-up covering period since 1961. The study area—Mid-Bohemia Pluton—is mostly granitoid with considerable geological breaks. The area of the study covers about 240 km^2^. The levels of radon concentration in the selected area are considerably higher than in the rest of the country. The study population includes inhabitants of the area (80 villages) who had resided there for at least three years, who were alive by the end of 1960 or were born later. The collected individual data in this study included date of birth, past residences, smoking habits, and housing characteristics. Data on 11,842 subjects were collected by trained interviewers who also installed radon detectors [4,5].

Information on vital status and dates of death were obtained from the Czech Population Registry at the Ministry of the Interior. Before 1982, the causes of death were obtained from local death registries and since 1982 from the Institute of Health Information and Statistics of the Czech Republic. Mortality data are available by the end of 2010.

The design of the present study is a nested case-control study, where for each case of lung cancer with smoking data, up to three or four controls were randomly selected from all cohort members matched by year of birth, age, gender, and the sub-cohort. Attained age in controls is considered at the year of death (index year) of the corresponding case and exposure and smoking data in controls reflect the situation at the index year. Data on smoking in the study were collected from subjects by personal interview, from medical records, and from relatives. The resulting residential nested case-control study in Mid-Bohemia Pluton (289 cases and 1,156 controls) was complemented by subjects from a large case-control hospital based study (Bulovka) conducted during 2000–2007 in Prague and adjacent regions in Central Bohemia [7]. A total of 81 cases and 243 controls (matched by age and gender) in this additional study were selected on condition of residing in the same dwelling for at least 30 years, where one-year radon measurements were realized.

### 2.2. Exposure Estimates

Details of exposure estimation in the occupational study have been described elsewhere [3,8]. Briefly, exposure estimates in the *S* study were derived from extensive measurements of radon already commencing in 1949. The concentrations of radon gas were converted to concentrations of radon decay products using measurements of equilibrium factors in mines when ventilation was not operated for 1 month. Each miner’s annual exposure to radon progeny was estimated combining measurement data with registered employment details, including duration of underground work at different shafts and job category. Missing exposure data in some years were extrapolated. In the *N* study the exposure estimates were based on personal dosimetric records, based on several thousand measurements per year and shaft. Some miners in both cohorts were also involved in non-uranium mines. Their exposures (substantially lower than those in uranium mines) were derived from radon measurement in non-uranium mines [9]. The exposure in the *L* sub-cohort were based on measurements of radon decay products since 1978. Before 1978, exposures were reconstructed according to ventilation conditions in the mines [6]. For historical reasons, the exposures are given in working level months (WLM) integrating concentration of radon decay product in working levels (WL) and duration of exposure in working months (170 h). One WL is equivalent to the concentration of short-lived radon decay products per liter of air that results in the emission of 130,000 MeV of alpha particle energy (1 WL = 3.7 kBq·m^−3^, 1 WLM = 629 h·kBq·m^−3^). 

The exposure assessment in the residential study was based on measurements of equivalent equilibrium concentrations of radon (radon progeny) in most houses (80%) of the study area. During the period 1991–1992, usually two passive track detectors (Kodak LR115, Lognes, France) in open configuration were installed for one year in two mostly occupied rooms of the house. In order to compare results to other residential studies, which are related to radon gas rather than radon progeny, values of equivalent equilibrium concentrations of radon were converted using 652 simultaneous measurements of radon progeny by passive track detectors and radon gas by electrets and CR-39 detectors (Pittsburgh, PA, USA) in closed configuration. All results are given in terms of radon gas concentrations. In the study area, the measurements were conducted in 2,154 houses (mean 499 Bq·m^−3^). Measured exposures were available for 72% of residential person-years. For houses in the study area that could not be measured (9%), community means were used instead of missing values (mean 551 Bq·m^−3^). Exposure estimates in residences outside the study area were derived from a large scale mapping of radon in the country conducted within the national Radon program. Concentrations corresponding to residences outside the study area (19% of respective residential person-years in relevant exposure window 5–34 years) were estimated by larger community means for inhabitants in neighboring four districts (mean 267 Bq·m^−3^) and by district means for the residences in other districts, where concentrations were usually much lower (mean 163 Bq·m^−3^). These different approaches reflected numbers of subjects residing in different areas [4,5].

### 2.3. Method of Analyses

The statistical assessment is based on conditional logistic regression with linear dependence of the relative risk (RR) on cumulated radon exposure (W), which was adjusted for smoking:

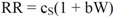
(1)
where coefficients c_S_ represent baseline relative risks at zero exposure in three smoking categories (never-. ex-, and current smokers) and b describes the linear trend between the risk and radon cumulated exposure (W) in WLM or average concentration in the previous 5–34 years in Bq·m^−3^. Time since exposure modifying effect is analyzed by two exposure windows:

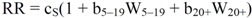
(2)


The exposure windows in the occupational study were limited to two periods 5–19 and 20+ years. This division follows results from one recent study of Czech uranium miners [3], where the temporal effect was investigated in details using 5 years exposure windows. The exposure periods 5–19, 20–29, and 30+ years were found to best describe the temporal modifying effect. In the present analyses these exposure windows were simplified to two windows 5–19 and 20+ as results from another earlier Czech study on radon and smoking gave practically similar results for exposure windows 20–29 and 30+ [10]. Exposure windows were not used in the residential study as the power to estimate such parameters is limited.

The multiplicative and additive interactions of radon exposure and smoking are evaluated using the so-called geometric mixture models, similarly like in the BEIR VI report [1]:

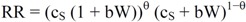
(3)


Here, the mixing parameter θ varies between 0 (additive model) and 1 (multiplicative model). The geometric mixture model with time since exposure windows is obtained from model Equation (3), where bW is replaced by time since exposure terms as in Equation (2):

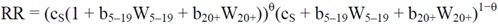
(4)


All models were estimated by maximum likelihood method using the Epicure software [11]. Confidence intervals for relative risks were calculated using the method of floating risk by Plummer [12]. The confidence levels of estimated parameters are 90% in order to correspond 1-sided statistical tests. The significance tests and confidence intervals are based on likelihood ratios.

### 2.4. Lifetime Risk Calculation

The probability of death from a specific cause at age *a* (during remaining life) is calculated as follows:

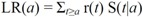
(5)
where S(*t*|*a*) is the probability of survival at age *a* to age *t*:

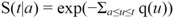
(6)
and q(*u*) is the total annual mortality at age *u* and r(*t*) is the cause specific mortality at age *t*. The probabilities of survivals can be obtained from life-tables routinely published by statistical offices. The lifetime risks were calculated for *a* = 0 until *t* = 100 using background specific lung cancer rates r_0_(*t*) in “non-exposed” population and with rates r_E_(*t*) in exposed populations. These rates are related as follows:

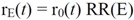
(7)
where RR(E) is the relative risk at exposure E, which may be one of the above relative risk models.

## 3. Results

### 3.1. Relative Risks in Case-Control Studies

In both cohorts, a total of 1,542 cases of lung cancer were observed by the end of 2010 (1,249 among miners and 293 in the residential study). Smoking data were collected for 1,029 and 289 cases and 2,648 and 1,156 controls, in the occupational and residential nested studies, respectively, see Table 1. In the present study, all analyses were carried out only for subjects with smoking information.

**Table 1:** Summary of nested case-control studies.

Study	Cases	Controls	Mean exposure among cases	Mean exposure among controls
*S* study	728	1,628	198 WLM	145 WLM
*N* study	212	739	9 WLM	8 WLM
*L* study	89	281	35 WLM	32 WLM
Entire occupational study	1,029	2,648	145 WLM	95 WLM
Mid-Bohemia Pluton	289	1,156	443 Bq·m^−3^	418 Bq·m^−3^
Bulovka	81	243	109 Bq·m^−3^	81 Bq·m^−3^
Entire residential study	370	1,399	370 Bq·m^−3^	359 Bq·m^−3^

Smoking categories in the present studies were considered as follows: never-smokers, ex-smokers (who quitted before 10 years or more) and other smokers including current smokers and ex-smokers who quitted before 10 years or less. These categories follow findings from preliminary risk calculations which showed that the relative risks among current smokers and ex-smokers (<10 years) were very similar. Numbers of cases and controls by smoking categories are given in Table 2.

**Table 2:** Summary of cases and controls by smoking.

	Occupational study		Residential study	
	cases	controls	cases	controls
Never-smokers	80	722	58	670
Ex-smokers (>10 y)	108	430	49	184
Other smokers	840	1,496	263	545
Total	1,029	2,648	370	1,399

Relative risks of lung cancer in relation to smoking and radon exposure categories (in terms of odds ratios) are given in Table 3. Here, odds ratios are given separately (crude OR) for smoking categories (ignoring radon exposure) and for categories of radon exposure (ignoring smoking). The risks are estimated also for categories of smoking with the adjustment to radon categories and similarly for radon categories adjusted to smoking (adjusted OR).

**Table 3:** Numbers of cases and controls and odds ratios in categories by smoking and radon exposure.

	Cases	Controls	Crude OR	90%CI	Adjusted OR	90%CI
**Miners**						
*Smoking*						
Never-smokers	80	722	1.00		1.00	
Ex-smokers (>10 y)	108	430	2.30	1.74–3.03	2.29	1.72–3.02
Other	840	1,496	5.98	4.78–7.49	5.79	4.61–7.27
*Radon exposure*						
<50 WLM	329	1,239	1.00		1.00	
50–99 WLM	163	499	1.64	1.28–2.12	1.70	1.31–2.22
100–199 WLM	249	539	2.47	1.90–3.21	2.46	1.87–3.23
200+ WLM	288	371	4.06	3.10–5.32	3.74	2.82–4.96
**Residents**						
*Smoking*						
Never-smokers	58	670	1.00		1.00	
Ex-smokers (>10 y)	49	184	3.96	2.65–5.93	4.00	2.68–5.99
Other	263	545	10.19	7.28–14.26	10.15	7.25–14.21
*Radon exposure* *						
<200 Bq/m^3^	85	311	1.00		1.00	
200–399 Bq/m^3^	118	484	1.52	1.01–2.29	1.41	0.91–2.17
400+ Bq/m^3^	167	604	1.84	1.20–2.82	1.76	1.11–2.80

* radon exposure is given in terms of average exposure in previous 5–34 years.

Smoking adjusted odds ratios in categories of radon exposure (in terms of multiplicative model where OR(smoking, radon) = OR(smoking) × OR(radon)) did not substantially change the effect from radon when smoking was ignored (Table 3). However, when the linear effect of cumulated radon exposure was evaluated separately for never- and ever-smokers, the risk coefficients (excess relative risk per unit exposure) were substantially (5 times) higher in never-smokers both in the occupational and residential studies (Table 4). The difference is significant in the occupational study (p = 0.033), but not in the residential study as the statistical power in this study is lower due to smaller numbers of cases and much lower cumulated exposures. In terms of radon progeny, mean exposure among miners was 69 MBq·m^−3^·h and among residents 30 MBq·m^−3^·h (assuming F = 0.4, 7,000 h at home annually). For the highest exposure categories, 300 WLM correspond to 189 MBq·m^−3^·h and 600 Bq·m^−3^ correspond to 50 MBq·m^−3^·h. In terms of intake (assuming breathing rate 1.2 m^3^/h in miners and 0.8 m^3^/h in residents) exposure 300 WLM corresponds to 227 MBq and exposure 600 Bq·m^−3^ corresponds to 40 MBq.

**Table 4:** Coefficients of excess relative risk per unit exposure by smoking categories

	Occupational study			Residential study		
	Cases	ERR/WLM	90%CI	Cases	ERR/100Bq·m^−3^	90%CI
Never smokers	80	0.049	0.010–0.179	58	0.73	0.02–1.9
Ever smokers	949	0.010	0.006–0.017	312	0.14	0.02–0.30
Smoking adjusted		0.014	0.009–0.023		0.14	0.03–0.39
Smoking ignored		0.013	0.007–0.019		0.12	0.02–0.32

**Figure 1:**
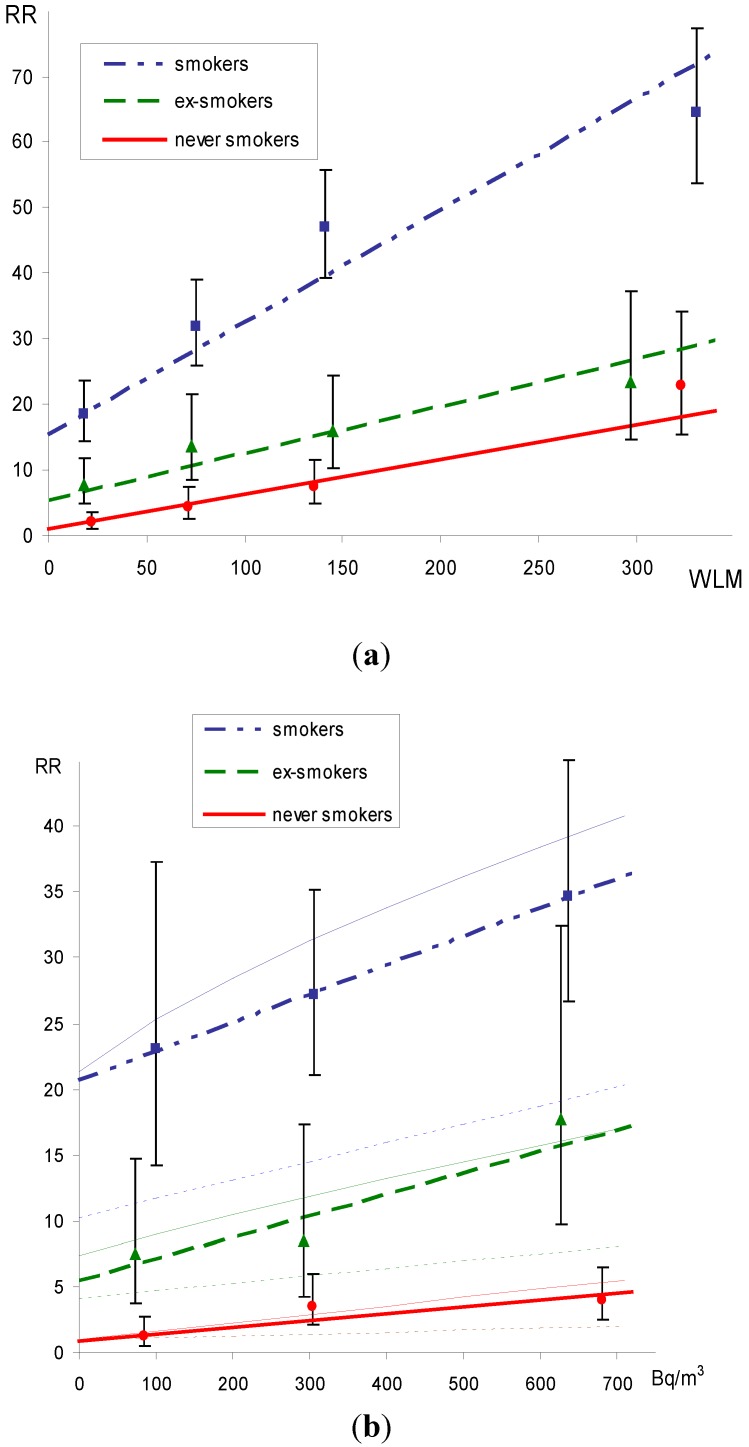
Relative risks (RR) by cumulated radon exposure and smoking categories. Panel (**a**) occupational study, panel; (**b**) residential study, including the multiplicative (dotted thin lines) and the best geometric mixture models (full thin lines).

Graphically, the dependence of the relative risks on cumulated exposure by smoking categories is given in Figure 1. 90%CI calculated using the method of floating risk by Plummer [12], RR scales adjusted for RR = 1 at zero exposure among never-smokers. 1 WLM corresponds to 629 kBq·m^−3^·h (Rn progeny) 30-year exposure at 1 Bq·m^−3^ corresponds to 84 kBq·m^−3^·h (Rn progeny), assuming F = 0.4 and 7,000 h at home annually.

### 3.2. Geometric Mixture Models

**Figure 2:**
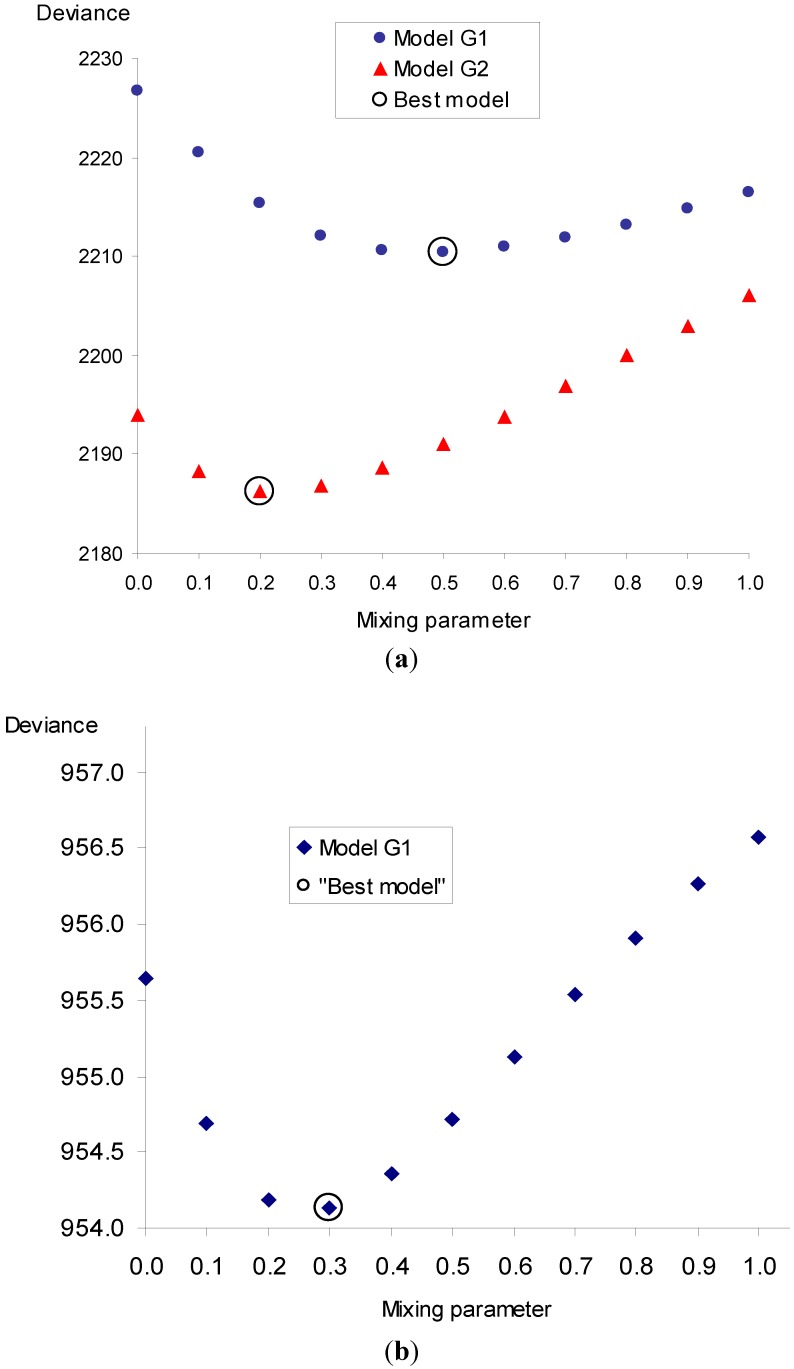
Dependence of deviances on mixing parameter θ in the occupational study for models G1 and G2 (panel **a**), and in the residential study for model G1 (panel **b**).

The above observed differences in the exposure-response relationship in smoking categories lead to the evaluation whether the combined effect of radon and smoking is additive or multiplicative. This issue is solved by the geometric mixture models with an additional parameter (θ), which include the additive (θ = 0) and multiplicative (θ = 1) models as special cases. The best model is the one with the lowest deviance as graphically presented in Figure 2. 

The results of fitting for different mixing parameters are given in Table 5. The best fit is achieved for θ = 0.2 in the occupational study (model G2) and for θ = 0.3 in the residential study (model G1). Both these values suggest that the best model of the combined effect from radon and smoking is closer to the additive model than to the multiplicative model. 

The dependence of the risk on residential exposure in Figure 1(b) is also given in terms of the fitted multiplicative and geometric mixture models. The main difference between the two models is in the estimates of risks related to smoking at zero exposure. The risks from smoking at zero exposure are substantially lower in the multiplicative model in comparison to the additive or the best geometric mixture models. The most likely reason is that the induced lung cancer cases from radon exposure do not contribute to spontaneous cases in the proportion usually observed in the smoking and non-smoking populations. In fact, the contribution from radon exposure is about the same in smokers and non-smokers, which corresponds more to the additive model.

The θ confidence interval for the occupational study corresponding to the G2 model is 0.05–0.48, suggesting a substantial difference from the multiplicative model. A θ confidence interval in the residential study is much wider because of lower numbers of cases and lower exposures and covers the entire interval 0–1. It should be noted that in the occupational study, the fit in terms of G2 model is substantially better than the G1 model (chi-sq = 24.1, p < 0.001).

**Table 5:** Parameters in fitted geometric mixture models is the occupational and residential studies for selected parameters θ.

**Occupational study**					
θ	Deviance		ERR/WLM	RR(S) *	90%CI
0.0	2,193.98	TSE 5–19	0.186		0.084–0.537
		TSE 20+	0.018		0.005 0.064
		ex-smoker		7.58	3.18–18.06
		smoker		24.33	10.18–58.15
0.2	1,661.73	TSE 5–19	0.149		0.074–0.338
		TSE 20+	0.020		0.007–0.058
		ex-smoker		5.98	3.27–10.95
		smoker		18.75	10.37–33.88
1.0	1,678.54	TSE 5–19	0.027		0.015–0.045
		TSE 20+	0.007		0.003–0.013
		ex-smoker		2.29	1.73–3.03
		smoker		5.81	4.62–7.30
**Residential study**					
θ	Deviance		ERR/100 Bq·m^−3^	RR(S) *	90%CI
0.0	955.64	TSE 5–34	0.578		0.080–168.3
		ex-smoker		8.88	3.19–24.72
		smoker		27.45	9.34–80.61
0.3	954.13	TSE 5–34	0.637		0.108–10.5
		ex-smoker		7.33	3.44–15.61
		smoker		21.38	9.93–46.03
1.0	956.58	TSE 5–34	0.138		0.026–0.390
		ex-smoker		4.11	2.74–6.15
		smoker		10.25	7.32–14.35

* RR(S) relative risk from smoking (at zero exposure) RR(never-smoker) = 1

### 3.3. Estimation of Induced Lifetime Risk from Indoor Radon

**Figure 3:**
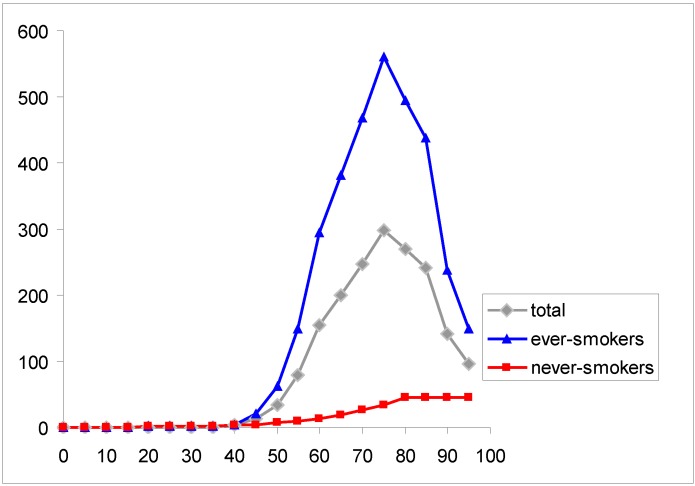
Age specific lung cancer rates (per 100,000) in the Czech Republic (men and women combined) and estimates of rates among smokers and non-smokers using mean prevalence (50%) of smokers and estimates in non-smokers according to Peto *et al.* [15].

**Figure 4:**
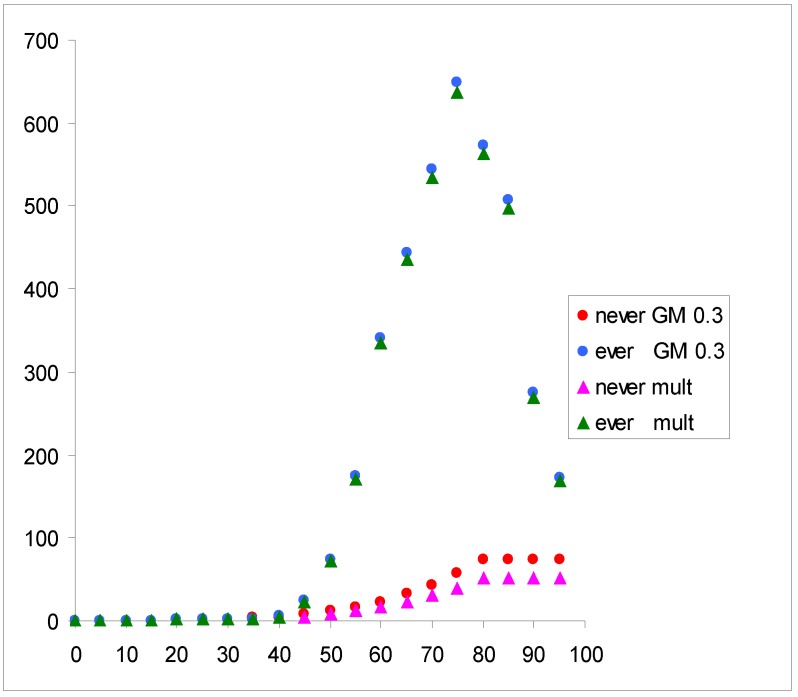
Age specific lung cancer rates (per 100,000) in a hypothetical populations of never- and ever-smokers exposed to 100 Bq·m^−3^ according to the resulting geometric mixture model (●) and the multiplicative model (▲).

The resulting estimates from indoor radon in geometric mixture models are translated into estimates of lifetime risks in the general population of the Czech Republic. Lifetime risks are calculated using mortality statistics in 2010 and estimated proportion of smokers (57% among men, 43% among women) in the country [13]. The lung cancer mortality rates in never-smokers are taken from [13] and the hypothetical mortality in smokers is calculated using the general mortality from lung cancer and average smoking prevalence (50%) in the Czech population, see Figure 3. The calculations of lifetime risk from lung cancer are based on life-tables in the Czech Republic in 2010 [14]. Lung cancer age specific rates in “non-exposed” population are taken according to the Czech population data in 2010 [13] and lung cancer rates in exposed population are calculated according to the multiplicative and geometric mixture models, Figure 4.

Results in terms of lifetime lung cancer numbers in 100,000 population are given in Table 6. As expected, numbers of lung cancers in smokers exceed numbers in non-smokers several times—about 14 times in non-exposed population and in exposed population according to the multiplicative model. However, the ratio of smoking to non-smoking lung cancers in exposed population is only about 10 according to the resulting geometric mixture model. Substantial difference between the two models is in numbers estimated in non-smokers—by a factor of nearly 5.

**Table 6:** Lung cancer lifetime risks (per 100,000) according to the multiplicative and best geometric mixture models in smoking and non-smoking populations (according to Czech statistics in 2010).

	Model	Non-smokers	Smokers	Total *
“non-exposed” population		666	9,165	4,915
exposed to 100 Bq·m^−3^	multiplicative	758	10,430	5,594
	geometric mixed (θ = 0.3)	1,090	10,655	5,872
induced cases	multiplicative	92	1,265	679
at 100 Bq·m^−3^	geometric mixed (θ = 0.3)	424	1,490	957

* total population with average smoking prevalence 50%.

## 4. Discussion

### 4.1. Data Collection

The results of this study rely on availability of smoking data. Among miners, particularly in the *S* sub-cohort, smoking data collection started in the mid-1990s, when about one-fourth of the cohort members were still alive. The investigation on smoking habits started firstly among cases and it was mostly based on medical files and information from relatives. The controls were randomly selected for each case with smoking data and data on controls were obtained in person if selected miners were alive and from relatives if selected controls deceased. This depended on the availability of addresses, which was more complicated for subjects who died long time ago. Therefore, earlier cases had less controls with smoking data than later cases. In the *N* and *L* sub-cohorts, smoking data were routinely collected during periodic medical checks in the 1970s and 1980s, when miners were asked to fill a simple questionnaire on number of cigarettes smoked per day and the year of cessation. These data were available for about 80% subjects. The controls selected in the *N* and *L* studies were asked again on their current smoking habits. Numbers of ex-smokers in the present study were generally low as the smoking status was considered at the index year, which was often before they stopped smoking.

### 4.2. Strengths and Limitations

The main strength of the present study is relatively low uncertainty of exposure estimates resulting from extensive measurements since nearly the beginning of the occupational study. The three occupational sub-studies are naturally different, namely as for the level of exposures and exposure duration. Exposure estimates in the *N* study are more precise then in the S study. This differences, however, is not reflected in the risk coefficients. The ERR/WLM coefficients in the multiplicative risk model—0.012 (*S*), 0.016 (*N*), and 0.012 (*L*) do not substantially differ (p = 0.97).

Limitations are in exposure data in the residential study. For obvious reasons, measurements of exposure in each year of relevant previous exposure period are not possible. Measurements in the present study were based on one-year measurements of radon, which are more reliable than short term measurements conducted for several months in most residential studies [16]. The effect from radon in residential studies is influenced by these uncertainties. The correction for uncertainty in the present study is planned in future, when the study of repeated measurements in residential study will be completed.

Limitations of the studies are in smoking data. In the occupational study, these data are based on personal interview or medical files, 60% and 63% among cases and controls, respectively. In the residential study of Mid-Bohemia Pluton, smoking data reported in person were 46% and 51% among cases and controls, whereas in the Bulovka study all smoking data were obtained in person. Smoking data not available in person or from medical files were obtained from relatives (mostly spouse, sometimes children). Generally, data obtained from relatives may not be quite exact as for the amount of cigarettes and years of quitting, but it is believed that the three smoking categories used in the models analyses are reliable. The estimated relative risks from smoking (at zero exposure) in the geometric mixture model (1, 6.0 18.8 and 1, 7.3, 21.4 for never-, ex-, and other smokers in the present occupational and residential studies) correspond to usual figures reported by other epidemiological studies (1, 7.5, 23.9) as summarized by Simonato *et al.* [17]. The evaluation of uncertainties in smoking data is planned in future, together with the uncertainties of exposure data.

Another limitation of the present study is a lack of smoking data among subjects who died long time ago as availability of relatives and their addresses was limited. For instance, for 102 cases who died before 1960, there was only 27 with smoking information. However, for later periods the coverage was at least 80%. The crude estimate of ERR/WLM in the entire cohort study was 0.010 (90%CI: 0.007–0.013) which is close to the value 0.013 (90%CI: 0.007–0.019), when smoking was ignored (Table 4). In the residential study, smoking data were available for 98% cases.

The evidence of an effect from residential radon is generally more difficult because of lower exposure and uncertainties in exposure estimates. The results of the present studies are in line with other residential studies. The excess relative risk per unit exposure (100 Bq/m^3^) in the present study, when smoking was adjusted for 0.14 is between the values 0.09 and 0.16 estimated in the European pooled study [16]. 

### 4.3. Comparison of Risk Coefficients in Relative Risk Models

The increased relative risk from radon exposure among non-smokers was consistently observed in the present study in all models. Seemingly surprising observations of higher excess relative risks per unit exposure in non-smokers do not mean that the absolute risks are higher because the baseline lung cancer risks are by about one order of magnitude higher. 

The relative risk coefficients (both ERR/WLM and ERR/Bq·m^−3^) when smoking is ignored is not substantially different from the one when smoking is adjusted for. The adjusted ERR/WLM in the occupational study is 0.014 and this risk coefficient is similar to that among smokers (0.010) as most cases are smokers. Similar results are seen in the present residential study. The ERR/Bq·m^−3^ was 0.14 among ever smokers and in the entire study when smoking was adjusted for.

The confounding effect of smoking in residential studies reported by Darby *et al* [16] generally reflects the association between radon exposure and smoking, *i.e.*, higher prevalence of smokers in low exposure groups, most likely because of the socio-economic status and type of housing. In the present Czech study, this association is relatively weak. In the occupational studies, the confounding effect by smoking was not observed in the present study, similarly as in other studies of uranium miners [18].

The model of relative risk with time since exposure specific ERR/WLM model (2) substantially improved the fit (p < 0.001) and deepened differences in smoking categories. In comparison to model (1), where the ratio of ERR/ WLM in never smokers and smokers was 5, this ratio of ERR/WLM corresponding to exposure window 5–19 was 9 [10], while the ERR/WLM from more distant exposures was substantially lower.

### 4.4. Comparison to Other Studies

The interaction between exposure to radon and smoking in six studies of miners were investigated by Lubin *et al.* and reported in BEIR VI [1]. Results for separate studies were consistent with a model intermediate between additive and multiplicative (mixing parameter between 0 and 0.7). Because of small numbers of cases particularly among non-smokers (64 cases), the additive and multiplicative models were not significantly different. Better fits were reported for the additive model in the China and Newfoundland studies, whereas better fits were observed for the multiplicative model in the Colorado, New Mexico and Sweden studies [1]. The joint analysis of all six studies resulted in a three times higher estimate of ERR/WLM (0.0103, 95%CI 0.002–0.057) among never smokers than among smokers (0.0034, 95%CI 0.0008–0.0170) [1]. In comparison to more different estimates in the present study, the uncertainty of estimates in the BEIR VI report is much higher despite large numbers of lung cancers (1,442 cases) observed in the joint study. Results by Placek *et al.* [6] based on follow-up up to 1990 of the *N* and *L* Czech cohorts found four times higher risk coefficients among non-smokers. 

In the present occupational study, the best estimate of mixing parameter (θ = 0.5, see Figure 2, model G1) was between the additive and multiplicative model in relation to cumulated exposure. Virtually the same findings (θ = 0.5) were reported by Leuraud in a large joint study of uranium miners (1,046 cases, 2,492 controls) in relation to cumulated exposure [18]. The mixing parameter in the present study, however, was substantially shifted (θ = 0.2) when time since exposure modifying factor was used. This approach could not been used in the residential study as the statistical power for two exposure windows is limited by smaller numbers of cases and substantially lower exposures in comparison to the occupational study. In the residential study, the best mixing parameter (θ = 0.3) is, however, close to the parameter estimated from the occupational study.

So far, in residential studies, the geometric mixture approach has not been used. The different estimates in non-smokers and smokers in the present residential study are in line with observations in other residential studies, where the relative risk per unit exposure is 2–3 times higher. For instance, in the joint European residential study [16] 2.2 times, in the joint North American residential study [19] 1.8 times, and in the joint China study [19] 2.6 times. These differences, however, were not statistically significant because of relatively low exposures and high uncertainties of exposures. The issue of additive and multiplicative effect of radon and smoking was recently investigated by Barros-Dios *et al.* [20]. They found an additive interaction when individuals were classified as exposed (>50 Bq·m^−3^) or non-exposed to residential radon and smokers in never- or ever-smokers. These results are supported by the present residential study. In comparison to never-smokers, the relative risk (OR) in smokers is 27.5 for exposures <200 Bq·m^−3^, whereas for exposure >200 Bq·m^−3^ the relative risk is only 8.0 .

Generally, the assessment of interactions between risk factors requires studies of higher statistical power than the evaluation of single risk factors. The strength of the study generally depends on the number of cases and on the levels of exposure. In studies of lung cancer risk from radon and smoking, the substantial issue is the level of radon exposure and its reliability.

In the BEIR VI report [1], potential reasons for interactions of smoking and radon exposure are given. Some of them include the impact of smoking on exposure, others exposure–dose relation and others dose-response relation in smokers and non-smokers as was reported by Baias *et al.* [21]. Although the study does not provide evidence relevant to each of the reasons, the probable explanations include differences in lung morphometry of target cells, thickness of the mucous layer and mucociliary clearance between smokers and non-smokers.

### 4.5. Lifetime Risk

The calculation of lifetime risk was realized in order to illustrate the impact of different models. This calculation demonstrated that current calculations of the risk, which are based on multiplicative effects between radon and smoking substantially underestimate the risk, particularly among non-smokers. As smoking prevalence in most countries decreases, this issue may be of importance, particularly in cost-benefit analyses.

## 5. Conclusions

The present results based on large numbers of lung cancer cases, particularly in the present occupational study, allowed us to estimate more precisely the combined effect of radon and smoking. The combined risk from radon and smoking is much closer to an additive then to a multiplicative interaction. In terms of lifetime risk estimates, the predictions from the resulting geometric mixture model are higher than the predictions from the multiplicative model.
